# Total and Inorganic Arsenic Contents in Some Edible Zingiberaceous Rhizomes in Thailand

**DOI:** 10.1155/2013/506389

**Published:** 2013-04-18

**Authors:** Chomkamon Ubonnuch, Suthep Ruangwises, Wandee Gritsanapan, Nongluck Ruangwises

**Affiliations:** ^1^Department of Pharmaceutical Chemistry, Faculty of Pharmacy, Mahidol University, 447 Sri-Ayutthaya Road, Ratchathewi, Bangkok 10400, Thailand; ^2^Department of Veterinary Public Health, Faculty of Veterinary Science, Chulalongkorn University, Henri Dunant Road, Bangkok 10330, Thailand; ^3^Department of Pharmacognosy, Faculty of Pharmacy, Mahidol University, Sri-Ayutthaya Road, Ratchathewi, Bangkok 10400, Thailand

## Abstract

The arsenic accumulation in rhizomes of Zingiberaceous plants was determined by atomic absorption spectrometry interfaced with hydride generation system (HG-AAS). The raw herbal materials, rhizomes, were collected from different regions of Thailand between December 2011 and January 2012. Six well-known Zingiberaceous plants, 16 samples from each and a total of 96 samples, were analyzed *Alpinia galanga* (Khaa), *Boesenbergia rotunda* (Kra-chaai), *Curcuma longa* (Khamin-chan), *Curcuma zedoaria* (Khamin-oi), *Zingiber cassumunar* (Plai) and *Zingiber officinale* (Ginger). Concentrations of total arsenic based on dry weight were 92.4 ± 9.2, 103.5 ± 20.8, 61.7 ± 12.5, 89.8 ± 17.5, 106.7 ± 19.5 and 69.3 ± 11.8 ng/g, respectively and inorganic arsenic were 48.8 ± 7.0, 66.3 ± 12.7, 25.5 ± 5.0, 38.7 ± 4.7, 71.2 ± 11.6, and 38.5 ± 5.5 ng/g, respectively. Among these, Plai and Kra-chaai exhibited the highest levels of total arsenic and inorganic arsenic accumulation that remind consumers to be aware of excess consuming of these rhizomes. On the contrary, the lowest value found in Khamin-chan indicating natural dietary supplements and herbal medicines comprising Kamin-chan are safe from arsenic poison. All investigated amounts of total and inorganic arsenic were much lower than limits recommended by Thai Food and Drug Administration.

## 1. Introduction 

Zingiberaceae, one of the largest families of the plant kingdom, is an important natural resource that provides many useful food products, spices, and traditional medicines to treat a variety of diseases [1, 2]. The consumption of herbal products for therapeutic purposes and to promote wellness is widely popular since people are greatly concerned about side effects of synthetic drugs [3]. Herbs are being increasingly used in the pharmaceutical industry as raw materials for the preparation of herbal medicines. The arsenic contamination of herbs may be due to environmental pollution [4]. Arsenic is widely distributed in the Earth's crust and present at an average concentration of 2 mg/kg. Around one-third of atmospheric flux of arsenic is of natural origin [5]. Arsenic is one of the first chemicals designated as a group 1 carcinogen [6] and well known to be poisonous to organisms [7]. The inorganic arsenic species (As(III) and As(V)) are the most toxic forms of arsenic present in food [8]. Previous studies have indicated that ingested inorganic arsenic is strongly associated with a wide spectrum of adverse health outcomes, primary cancers, and other chronic diseases [9]. The primary route of arsenic exposure for the general population is via ingestion. The daily intake of total arsenic from food and beverages is generally in the range of 20–300 *μ*g/day [10]. Furthermore, Joint Food and Agriculture Organization/World Health Organization Expert Committee on Food Additives note that benchmark dose lower confidence limit for a 0.5% (BMDL 0.5) of inorganic arsenic in human was 3.0 *μ*g/kg body weight per day [11]. 

Root or rhizome is a part of the plant that has high opportunity to contaminate with arsenic. Current research demonstrated that plants absorbed heavy metals from soil [12, 13]. The contaminants and residues of toxic metal arsenic may cause harm to the consumers of herbal medicines. Plants that grow in an arsenic-affected area may have a high level of arsenic. The transfer of arsenic from soils to plants might be a key step in the route of arsenic entry into human body [14, 15]. Experimental data had shown that a variety of vegetable crops accumulate arsenic by root uptake from soil deposited on the leaves [16], and Gulz et al. [17] reported several edible plants grown in contaminated soils that accumulated high levels of arsenic. Arsenic species from soil can enter into edible tissues through absorption. The WHO [5] determined that arsenic concentration in plants grown in soils without arsenic-containing pesticides varied from 0.02 to 5 mg/kg (dry weight), and in the arsenic soil contamination indicated root can contain higher levels of arsenic than other parts of plant. 

A previous study about rhizome of *Zingiber officinale* (Ginger) shows that arsenic level varied from not detected to 0.13 *μ*g/L [18]. Other arsenic contamination reports of Karadas and Kara [19] show that arsenic levels in cumin and turmeric were 174 ± 14 and 39 ± 5 ng/g, dry wt, respectively. Vegetable crops in the contaminated region were found high in arsenic level (dry wt) in *Arum* ranged from 74.3 to 89.2 mg/kg, in cabbage from 27.12 to 39.39 mg/kg and in pumpkin from 17.28 to 22.05 mg/kg [20]. Baroni et al. [21] analyzed sixty-four plant species, and the highest arsenic contents were found in roots of *Phragmites australis* (688 mg/kg). Roychowdhury et al. [22] investigated high arsenic levels in cumin and turmeric (47.86–209.75 and 297.33–280.9 ng/g, res.) Moreover, dry Ginger exhibited mean arsenic content of 77.9 ± 8.7 ng/g [14]. In general, the highest concentration of arsenic was found in plant roots, the intermediate level in vegetative tissues (leaves and stems), and the lowest level in reproductive tissue (fruits and seeds) [23, 24]. Plants absorb arsenic fairly easily, so that high-ranking concentration may be present. Among many of public researches, most studies had focused on foods and not much on the information that was available on plants especially in the part of rhizomes although it was a high opportunity for arsenic accumulation. For this reason, well-known rhizomes of Zingiberaceae family that are used as food, dietary supplements and alternative medicines in Thailand, that is, *A*.* galanga*, *B*.* rotunda*, *C*.* longa*, *C. zedoaria*, *Z. cassumunar, *and *Z. officinale,* were interesting to investigate their total and inorganic arsenic concentrations.

## 2. Materials and Methods

### 2.1. Chemicals

Standard reference material (SRM) 1568a (rice flour) was purchased from the National Institute of Standards and Technology (Gaithersburg, MD, USA). Nitric acid (HNO_3_) and hydrochloric acid (HCl) were purchased from Merck Chemicals (Darmstadt, Germany); dimethylarsinic acid (DMA), hydrazine sulfate, hydrobromic acid, and other chemicals were obtained from Sigma-Aldrich (St. Louis, MO, USA). All standard solutions, reagents, and samples were prepared using deionized water (18 MΩ cm) throughout the study. To remove possible arsenic residue contamination, all glasswares were washed thoroughly with tap water, air-dried, soaked in 10% (v/v) HNO_3_ for 20–24 h, and washed three times with deionized water.

### 2.2. Sample Collection

Six kinds of plants in the family Zingiberaceae of which rhizomes are widely used for consumption and medication purposes were selected. They were *Alpinia galanga* (L.) Willd. (Khaa), *Boesenbergia rotunda* (L.) Mansf. (Kra-chaai), *Curcuma longa* (L.) (Khamin-chan), *Curcuma zedoaria* (Berg.) Roscoe (Khamin-oi), *Zingiber cassumunar* Roxb. (Plai), and *Zingiber officinale* Roscoe (Ginger). Sixteen samples of each rhizome species were collected during December 2011 and January 2012 from eight provinces (Chiang Rai, Lampang, Khon Kaen, Ubon Ratchathani, Ratchaburi, Samut Prakan, Krabi, and Songkhla) in north, northeast, central and south Thailand. Totally, 96 samples were collected for investigation. These collections were grown in urban and agricultural fields. Growing period was around two years and harvesting time from December to January. The noticed appearances of mature rhizome were strong flavor and pungent odor. The samples were identified by Dr. W. Gritsanapan, and the voucher specimens (AG111201-16, BR120101-16, CL111201-16, CZ111201-16, ZC120101-16, and ZO120101-16) were deposited at Department of Pharmacognosy, Faculty of Pharmacy, Mahidol University, Thailand.

### 2.3. Sample Preparation

Rhizome samples were washed, cleaned, air-dried, and sliced. Freeze drying is used to dry sliced rhizomes to keep them in a stable condition. The dried samples were grinded into powder with a porcelain mortar and pestle and passed through a fine mesh sieve. Powder of lyophilized samples was kept in air tight containers at 4°C and protected from light until analysis. The moisture contents were calculated using weights of samples before and after lyophilization.

### 2.4. Determination of Total Arsenic

The lyophilized sample preparation for determination of total arsenic was performed by acid digestion procedure described by Muñoz et al. [25]. An accurate weight (0.5 ± 0.01 g) of each lyophilized sample was mixed with 1 mL of an ashing suspension (20% (w/v) Mg(NO_3_)_2_·6H_2_O and 2% (w/v) MgO in water) and 5 mL of 50% (v/v) HNO_3_. The mixture was evaporated on a hot plate to dryness and mineralized at 450°C in a furnace. The resulting white ash was dissolved in 5 mL of 6 N HCl and 5 mL of a freshly prepared reducing solution (5% (w/v) KI and 5% (w/v) ascorbic acid). The solution was left for 30 min, and then 10 mL of 50% (v/v) HCl was added to the solution. The solution was filtered through a Whatman no. 1 filter paper into a 25 mL volumetric flask and adjusted to volume with 50% (v/v) HCl. The resulting solution was used for the determination of total arsenic. Duplicate analyses were performed for individual samples.

### 2.5. Determination of Inorganic Arsenic

Inorganic arsenic was determined by the nonchromatographic method described by Muñoz et al. [25]. An accurate weight (1.0 ± 0.01 g) of each lyophilized sample was placed in a 50 mL screw-capped centrifuge tube; 4.1 mL of water was added to the sample and mixed until completely moistened. In order to hydrolyze As(III) from the thiol groups of proteins, 18.4 mL of concentrated HCl was added to the moistened sample, shaken for 1 h, and left overnight (12 to 15 h). A reducing agent (1 mL of 1.5% (w/v) freshly prepared hydrazine sulfate and 2 mL of hydrobromic acid) was added to the sample tube and vortexed for 2 min. For extraction of inorganic arsenic, 10 mL of chloroform was added to the tube, vortexed for 3 min, and inverted for 1 min. To break the emulsion formed during the extraction, the tube was centrifuged at 1,000 ×*g* (measured in gravity × force or *g*-force) for 10 min using an Eppendorf bench top centrifuge 5810 (Hamburg, Germany). The chloroform phase was aspirated into another centrifuge tube. The extraction process was repeated twice. To separate some residues from the extraction, the combined chloroform phase was filtered through a syringe filter with a 25 mm PTEF membrane, pore size 0.45 *μ*m (Chrom Tech, Apple Valley, MN, USA), into another tube. Inorganic arsenic in the chloroform phase was back-extracted into an aqueous phase with 10 mL of 1 N HCl and centrifuged at 1,000 ×*g* for 10 min. The aqueous phase was aspirated into a beaker. The chloroform phase was extracted one more time. The amount of inorganic arsenic in the combined acidic aqueous phase was quantified as described in Section 2.4, with the addition of 2.5 mL of the ashing suspension and 10 mL of 50% (v/v) HNO_3_. All samples were analyzed for inorganic arsenic in duplicate.

### 2.6. Instrumentation

A PerkinElmer (Waltham, MA, USA) AAnalyst 300 atomic absorption spectrometer (Norwalk, CT, USA) interfaced with an AS-90 autosampler and a FIAS-400 flow injection system was used to determine total and inorganic arsenic concentrations in the final solutions. The atomic absorption spectrophotometric conditions were as follows: wavelength, 193.7 nm; slit width, 0.70 nm; EDL current, 380 mA; loop sample, 0.5 mL. The hydride generation conditions were as follows: quartz cell, 16 cm path length, 90.7 cm i.d., electrothermal heating, cell temperature, 900°C, carrier gas (argon) flow rate, 50–100 mL/min, reducing agent (0.2% (w/v) NaBH_4_ in 0.05% (w/v) sodium hydroxide solution) flow rate, 5–7 mL/min, and HCl flow rate, 9–11 mL/min [26].

### 2.7. Determination of Limit of Quantification

The Q2B analytical procedure described by the US FDA was used for determination of the limit of quantification (LOQ). Mixtures of lyophilized samples of all six species with equal weights were used for the determination of the limit of quantification (LOQ) of the method. For the determination of the LOQ for total arsenic, samples were fortified with a standard arsenic mixture (As(III) : dimethylarsinic acid (DMA), 1 : 1 (w/w)) equivalent to total arsenic at concentrations of 250, 500, 1,000, and 2,500 ng/g; blank samples were not fortified with arsenic. All samples were analyzed in duplicates. A total of twelve regression lines (six regression lines each for intraday and interday analyses) were obtained by the least-square linear regression analyses of the residual peak heights of standard total arsenic versus fortified total arsenic concentrations. The residual peak heights were peak heights of total arsenic-fortified samples minus that of blank sample. The LOQ of the method was calculated using the equation LOQ = 10*σ*/*S*, where *σ* is the standard deviation of *y*-intercepts and *S* is the slope of linear regression analysis [27].

For the determination of the LOQ for inorganic arsenic, samples were fortified with an inorganic arsenic mixture (As(III) : As(V) 1 : 1 (w/w)) at concentrations of 50, 100, 500, and 1,000 ng/g; blank samples were not fortified with inorganic arsenic. Duplicate analyses were performed for individual samples. A total of twelve regression lines (six regression lines each for intraday and interday analyses) were obtained by the least-square linear regression analyses of the residual peak heights of standard inorganic arsenic versus fortified inorganic arsenic concentrations. The residual peak heights were the peak heights of fortified samples of inorganic arsenic minus the average peak height of blank sample. The LOQ of the method was calculated using the equation LOQ = 10*σ*/*S*, where *σ* is the standard deviation of *y*-intercepts and *S* is the slope of linear regression analysis [27].

### 2.8. Quality Assurance

The accuracy of determination of total arsenic was assessed by analyzing SRM 2568a (rice flour), because no commercial rice standard reference materials for inorganic arsenic are available. The amount of inorganic arsenic in SRM 1568a (rice flour) was determined and compared with the values previously reported.

### 2.9. Statistical Analysis

One-way analysis of variance and Tukey's test using the SPSS Statistics version 17.0 software were performed to determine differences in concentrations of total arsenic, inorganic arsenic (both on wet weight and dry weight basis), and percentages of inorganic arsenic with respect to total arsenic of six rhizome types. A significance level of *P* < 0.05 was accepted for all comparisons. 

## 3. Results and Discussion 

The calculation for LOQs was based on the standard deviation of *y*-intercepts of the linear regression analysis (*σ*) and the slope (*S*) by using the equation LOQ = 10*σ*/*S* [27]. The LOQs for total and inorganic arsenic in rhizome samples were 19.7 and 15.7 ng/g, respectively. Concentrations of total arsenic and inorganic arsenic found in SRM 1568a (rice flour) were 283 ± 34 ng/g (*n* = 10, reference value of 290 ± 30 ng/g) and 102 ± 3.7 ng/g (*n* = 10), respectively [26]. The concentration of inorganic arsenic was in agreement with previous reports of 111 ± 6 ng/g [25] and 111 ± 3 ng/g [28], which was analyzed by the same method. The accuracy and precision for the determination of total and inorganic arsenic in rhizome samples fortified with arsenic mixture at four concentrations are shown in Table 1. The accuracy was assessed as percent recovery from the analysis of fortified arsenic mixture in the rhizome samples. The average recoveries across the four concentrations of fortified arsenic mixtures were 95.6% and 95.4% for total and inorganic arsenic, respectively. The precision of the method expressed as percentage of relative standard deviation (% RSD) was calculated with the equation % RSD = 100SD/ $\overset{-}{x}$, where SD is the standard deviation and $\overset{-}{x}$ is the mean of arsenic concentrations recovered from the arsenic-fortified samples. The % RSD ranged from 1.7 to 5.0 for total arsenic and from 1.1 to 5.5 for inorganic arsenic.

Table 2 summarizes concentrations of total arsenic, inorganic arsenic, and percentages of inorganic arsenic with respect to total arsenic in rhizomes of six plants in Zingiberaceae family collected from four regions of eight areas in Thailand. The highest content of inorganic arsenic was found in Plai (71.2 ± 11.6 ng/g, dry wt). The inorganic arsenic content in six dried rhizomes was expressed within the range of 20.4 to 92.8 ng/g, total arsenic levels ranged from 42.5 to 145.1 ng/g, and the percentages of inorganic arsenic with respect to total arsenic ranged from 28.5 to 83.2 ng/g.

Zhao et al. [14] determined concentrations of total arsenic in dried Ginger (*n* = 3) from China. Average concentration was 77.9 ng/g, whereas the value in this study was 69.3 ng/g. The indicated concentration may imply that the growth environments and soil condition for growing Ginger in Thailand (69.3 ± 11.8 ng/g) were safer than those in China (77.9 ± 8.7 ng/g) [14]. The study of the arsenic-affected area in India reported higher levels of arsenic in fresh cumin and turmeric powder than our study. They reported that arsenic level ranged from 47.86 to 209.75 ng/g and from 297.33 to 280.9 ng/g, respectively [22]. Our study found that the total arsenic levels in Khamin-chan (wet wt and dry wt) ranged from 5.8 to 21.3 ng/g and from 42.5 to 87.2 ng/g, respectively. These results clearly showed that the high amount of arsenic deposited in rhizomes is according to soil location.

In six plants of Zingiberaceae family in this study, the average concentrations of inorganic arsenic in the roots of *C. longa* and *C. zedoaria* were 4.3 ± 1.5 and 6.2 ± 1.5 ng/g (wet wt), respectively. The inorganic arsenic contents in two *Curcuma* species were not statistically different (*P* > 0.05) but they were significantly different (*P* < 0.05) from *Zingiber* genus. The inorganic arsenic contents in *Z. cassumunar* and *Z. officinale* were 11.7 ± 4.5 and 4.4 ± 1.0 ng/g (wet wt), respectively. The well-known species of *Alpinia* genus, *A. galangal*, showed the concentration (wet wt) of total arsenic (15.9 ± 5.1 ng/g) and inorganic arsenic (8.4 ± 3.0 ng/g). Finally, Kra-chaai in *Boesenbergia* genus contained the total arsenic level of 20.8 ± 4.6 ng/g, the inorganic arsenic level of 13.4 ± 3.1 ng/g, and the percentage of inorganic arsenic of 64.4%. 

In this study, the total arsenic concentrations in fresh rhizomes of Kra-chaai, Plai, Khaa, Khamin-oi, Khamin-chan, and Ginger ranged from 11.5 to 27.3, 7.9 to 26.8, 10.0 to 28.3, 6.9 to 21.8, 5.8 to 21.3, and 4.8 to 13.7 ng/g, respectively. These values are in accordance with the fresh vegetable arsenic concentration reported that ranged from 0 to 195 ng/g. Other edible roots, such as carrot and potato were reported high levels of arsenic at 195 and 103 ng/g, respectively [29]. Muñoz et al. [25] reported that the total arsenic level for vegetable group ranged from 8 to 604 ng/g, and the total and inorganic arsenic concentrations in edible roots or rhizomes were higher than other organs of herbs. 

Of the six medicinal rhizomes of Zingiberaceae family analyzed in this study, both of Kra-chaai and Plai exhibited high mean levels (wet weight) of total arsenic (20.8 ± 4.6 and 17.5 ± 6.6 ng/g), inorganic arsenic (13.4 ± 3.1 and 11.7 ± 4.5 ng/g), and percentage of inorganic arsenic (64.4 and 67.4%), respectively. A possible explanation is that these high levels were related to their arsenic accumulative capacity in the rhizosphere soils associated with each species. In contrast, the low levels of inorganic arsenic in Khamin-chan, Khamin-oi, and Ginger (wet weight) ranged from 2.6 to 8.2, 2.5 to 8.4, and 2.6 to 6.0 ng/g, respectively, whereas the average percentages of inorganic arsenic were 41.8, 44.5, and 56.9, respectively. Form the statistical analysis, comparative contents of total arsenic (ng/g, dry wt) among six species, Khaa, Kra-chaai, Khamin-oi, and Plai were significantly different from Khamin-chan and Ginger.

From our study, the total arsenic level in ninety-six samples complied with Thai regulatory limit (2 *μ*g/g) and national limit for arsenic in herbal medicines (4 *μ*g/g) [30–32].  Table 3 shows a comparison of total arsenic level in some rhizomes or roots from various countries. Only a few studies were reported. Further investigations should be performed on other botanical rhizomes with incidence of high arsenic accumulation to crucially guarantee that plants are safe for dietary supplements for health and well-being. 

## 4. Conclusion

All investigated amounts of total arsenic concentrations meet the requirements at a national level. Among six species were grown on urban and agricultural areas. We found that dried root of *Z. cassumunar* showed a higher level of inorganic arsenic than other species. Arsenic accumulation in rhizomes may be an important risk factor that needed to be taken into account. The results will be valuable for preliminary risk assessment in raw materials of natural products. Therefore, long-term consumption of herbal products that comprised some particular Zingiberaceous rhizomes might cause adverse health effects.

## Figures and Tables

**Table 1:** Accuracy and precision in the determination of total and inorganic arsenic in Zingiberaceae rhizomes.

Arsenic added (ng/g)	Intraday (*n *= 6)			Interday (*n* = 6)		
	Found (ng/g) mean ± SD	% RSD^a^	Recovery (%)	Found (ng/g) mean ± SD	% RSD^a^	Recovery (%)
Total						
250	244.1 ± 8.5	3.5	97.6	237.4 ± 6.7	2.8	94.9
500	473.1 ± 13.5	2.9	94.6	477.8 ± 21.2	4.4	95.6
1,000	944.7 ± 32.6	3.5	94.5	972.2 ± 48.7	5.0	97.2
2,500	2,366.3 ± 41.5	1.8	94.7	2,382.0 ± 40.6	1.7	95.3
Inorganic						
50	47.3 ± 2.6	5.5	94.7	47.7 ± 2.6	5.4	95.3
100	98.1 ± 4.8	4.9	98.1	97.9 ± 3.4	3.4	97.9
500	469.8 ± 25.2	5.4	94.0	473.0 ± 21.9	4.6	94.6
1,000	943.7 ± 13.5	1.4	94.4	940.7 ± 10.7	1.1	94.1

^a^% RSD: percent relative standard deviation.

**Table 2:** Total arsenic, inorganic arsenic, and percentage of inorganic arsenic with respect to total arsenic for rhizomes of six plants in the Zingiberaceae family collected from four regions in Thailand (north, south, central, and northeast)^a^.

Species	*n *	Total arsenic^a^ (ng/g)		Inorganic arsenic^a^ (ng/g)		% Inorganic arsenic^b^
		Wet wt	Dry wt	Wet wt	Dry wt	
*Alpinia galanga *	16	15.9 ± 5.1 A	92.4 ± 9.2 A	8.4 ± 3.0 A	48.8 ± 7.0 C	53.2 ± 8.2 A
(Khaa)		(10.0–28.3)	(74.7–107.2)	(4.5–15.6)	(39.3–65.1)	(38.9–69.6)
*Boesenbergia * *rotunda *	16	20.8 ± 4.6 A	103.5 ± 20.8 A	13.4 ± 3.1 B	66.3 ± 12.7 A	64.4 ± 4.9 A
(Kra-chaai)		(11.5–27.3)	(80.3–140.3)	(6.4–17.6)	(47.3–92.8)	(56.1–74.3)
*Curcuma longa *	16	10.3 ± 3.8 A	61.7 ± 12.5 B	4.3 ± 1.5 A	25.5 ± 5.0 D	41.8 ± 6.2 A
(Khamin-chan)		(5.8–21.3)	(42.5–87.2)	(2.6–8.2)	(20.4–36.0)	(30.5–53.5)
*Curcuma zedoaria *	16	14.5 ± 4.6 A	89.8 ± 17.5 A	6.2 ± 1.5 A	38.7 ± 4.7 B	44.5 ± 10.0 A
(Khamin-oi)		(6.9–21.8)	(55.0–126.8)	(2.5–8.4)	(24.4–44.6)	(28.5–72.8)
*Zingiber cassumunar *	16	17.5 ± 6.6 A	106.7 ± 19.5 A	11.7 ± 4.5 B	71.2 ± 11.6 A	67.4 ± 7.5 A
(Plai)		(7.9–26.8)	(73.6–145.1)	(5.6–20.2)	(52.7–92.1)	(54.6–83.2)
*Zingiber officinale *	16	7.9 ± 2.2 A	69.3 ± 11.8 B	4.4 ± 1.0 A	38.5 ± 5.5 B	56.9 ± 11.8 A
(Ginger)		(4.8–13.7)	(43.3–86.4)	(2.6–6.0)	(30.1–48.5)	(42.4–82.6)

^a^There were 96 samples in total, 16 of each of the six species. Values are mean ± SD; numbers in parentheses are ranges.

^b^% inorganic arsenic = (concentration of inorganic arsenic × 100)/concentration of total arsenic.

A, B, C, and D values in the same column followed by different letters denote significant differences (*P* < 0.05).

**Table 3:** Concentrations of total arsenic in roots or rhizomes from various countries.

Species	Location	Total arsenic concentration (ng/g)	Reference
Cumin^a^	Murshidabad district, West Bengal, India	47.86–209.75	[15]
Turmeric powder^a^	Murshidabad district, West Bengal, India	297.33– 280.9	[15]
*Curcuma l* *on* *ga* *e* ^b^	Thailand	61.7 ± 12.5^c^	Present study
Dry ginger^a^	Beijing, China	77.9 ± 8.7^c^	[5]
*Zingiber o* *ff* *ic* *in* *al* *e* ^b^	Thailand	69.3 ± 11.8^c^	Present study
*Isatis * *in* *d* *ig* *ot* *ic* *a* ^b^	Anguo city, Hebei, China	137.4 ± 0.0^c^	[3]
*Atractylodes m* *ac* *ro* *ce* *ph* *al* *a* ^b^	Anguo city, Hebei, China	371.9 ± 0.0^c^	[3]
*Salvia m* *il* *ti* *or* *rh* *iz* *a* ^b^	Anguo city, Hebei, China	278.9 ± 40.2^c^	[3]
*Saposhnikovia divaricata* ^b^	Anguo city, Hebei, China	175.8 ± 23.9^c^	[3]
*Astragalus m* *em* *br* *an* *ac* *eu* *s* ^b^	Anguo city, Hebei, China	140.3 ± 9.5^c^	[3]
*Aster t* *at* *ar* *ic* *us* ^b^	Anguo city, Hebei, China	525.5 ± 103.9^c^	[3]
*Anemarrhena a* *s* *ph* *o* *de* *lo* *i* *de* *s* ^b^	Anguo city, Hebei, China	216.2 ± 31.6^c^	[3]
*Trichosanthes k* *ir* *il* *ow* *ii* ^b^	Anguo city, Hebei, China	184.4 ± 6.9^c^	[3]
*Alpinia * *ga* *l* *an* *ga* ^b^	Thailand	92.4 ± 9.2^c^	Present study
*Boesenbergia r* *ot* *u* *nd* *a* ^b^	Thailand	103.5 ± 20.8^c^	Present study
*Curcuma z* *ed* *o* *ar* *i* *a* ^b^	Thailand	89.8 ± 17.5^c^	Present study
*Zingiber c* *as* *su* *m* *un* *ar* ^b^	Thailand	106.7 ± 19.5^c^	Present study

^a^Species not specified.

^b^The roots or rhizomes of plants can be used as botanical products.

^c^Value as mean ± SD.
